# Prevalence, impacts and medical managements of premenstrual syndrome among female students: cross-sectional study in college of health sciences, Mekelle University, Mekelle, Northern Ethiopia

**DOI:** 10.1186/1472-6874-14-52

**Published:** 2014-03-29

**Authors:** Fikru Wakjira Tolossa, Mebratu Legesse Bekele

**Affiliations:** 1 Dembidolo Hospital, Wellega, Western Ethiopia; 2 College of Health Sciences and Medicine, Wolaita Sodo University, Wolaita Sodo, P.O.Box: 138, Southern Ethiopia

**Keywords:** Prevalence, Impacts, Medical management, PMS, Female students

## Abstract

**Background:**

Premenstrual syndrome (PMS) is used to describe physical, cognitive, affective, and behavioral symptoms that occur cyclically during the luteal phase of the menstrual cycle and resolve quickly at or within a few days of the onset of menstruation. The primary aim of the study was to assess the prevalence, impacts and medical managements of PMS on female medical students of Mekelle University College of Health Sciences.

**Methods:**

A cross-sectional study was conducted among systematically selected female students of Mekelle University College of Health Sciences, Mekelle town, northern Ethiopia from March to April 2013. A structured and pretested self-administered questionnaire was employed for data collection. The collected data were analyzed using the Statistical Package for the Social Sciences, SPSS Inc., Chicago, IL (SPSS version 16). The criteria proposed by the Diagnostic and Statistical Manual of Mental Disorders, fourth edition, text revision (DSM-IV TR) were used to diagnose PMS.

**Result:**

From the total population size of 608; a sample size of 258 was drawn. Age of the study participants ranged from 18 to 25 years, with mean age of 20.86 ± 1.913 years. Among the participants, 144(83.2%) have had at least one PM symptoms with their menstrual period. The prevalence of PMS according to DSM-IV was 37.0%. About 49(28.3%) reported frequent class missing, 17(9.8%) exam missing, 14(8.1%) low grade scoring and 3(1.7%) of them reported withdrawal from their learning associated with their PMS. Only 83(48.0%) participants sought medical treatment for their PMS. The treatment modalities used were pain killers, 63(36.4%), hot drinks like coffee and tea, 13(7.5%), and massage therapy and exercise, 7(4.0%). Binary logistic regression analysis revealed average length of one cycle of menstruation (COR = 0.20(0.070-0.569) and academic performance impairment (AOR = 0.345(0.183-0.653) were significantly associated with the diagnosis of PMS and use of PMS treatments respectively.

**Conclusions:**

Our study revealed a high prevalence and negative impact of PMS on students of Mekelle University. Therefore, health education, appropriate medical treatment and counseling services, as part of the overall health service, should be availed and provided to affected women.

## Background

Premenstrual syndrome (PMS) is used to describe physical, cognitive, affective, and behavioral symptoms that occur cyclically during the luteal phase of the menstrual cycle and resolve quickly at or within a few days (7 to 14 days) of the onset of menstruation [1,2]. Premenstrual tension is the lay term that is used for PMS; premenstrual dysphoric disorder (PMDD) is the extreme, predominantly psychological, end of the PMS spectrum. The identified core symptoms of PMS and PMDD are: anxiety/tension, mood swings, aches, appetite/food cravings, cramps, and decreased interest in activities. These symptoms fall into three domains: emotional, physical, and behavioral [2-5].

The evolution of diagnostic criteria for PMS has a confusing and controversial history that has led to frustration among scholars and caregivers who are unclear of what symptoms constitute either disorder. The Diagnostic and Statistical Manual of Mental Disorders, fourth edition, text revision (DSM-IV TR) classified PMS as a mental disorder and termed it the ‘premenstrual dysphoric disorder [6]. Today, the tenth revision of the International Classification of Diseases (ICD-10) places PMS under “Diseases of the genitourinary system: Pain and other conditions associated with female genital organs and menstrual cycle” and labels it as Premenstrual Tension Syndrome [7].

Epidemiological surveys have estimated that the frequency of PMS symptoms is quite high about 80-90%, and about 5% of women experience severe symptoms that the symptoms interfere with their daily activities. Another finding also reported that at least 25% of all adolescent girls aged 14–15 years experienced the incidence of Premenstrual Syndromes (PMS) [3]. Among the gynecological problems, menstrual problems are said to be the major ones especially among adolescent females [8]. But, the true prevalence of PMS is difficult to determine because of self-treatment, difference in availability and access to medical care, definition & diagnostic criteria and cultural practices [9]. Studies in different countries indicated that PM symptoms are more common and more sever among high-level educated women than non- educated women with a possible association of stress with PMS. In the community studies performed, the prevalence of PMS was found between 5.9% and 90% in the women in the age group of 15–49 and between [6,10-12]. In Ethiopian context, there is few published data on PMS, as this disorder was not taken as public health problem; thus, prevalence of PMS is not known at national level [6].

PMS is related to high suicide and accident rates, employment and school absentee rates, poor academic performance and acute psychiatric problems. PMS is one of the factors that make women more susceptible than men to depression, particularly during periods of rapid fluctuation of gonadal hormones, such as premenstrual, postpartum and the climacteric. Studies in different countries indicated that PM symptoms are more common and more severe among high-level educated women than non-educated women with a possible association of stress with PMS [6,7,12-15]. Furthermore, child disturbance and family violence in the families of the patients with PMS were reported. In this sense, PMS may affect not only the individual, but also her family and the community [10].

The pathophysiology of PMS remains unknown, complex and multifactorial and yet to be fully clarified and may include the effect of progesterone on neurotransmitters such as serotonin, opioids, catecholamine and GABA, increased prolactin level or increased sensitivity to the effect of prolactin, insulin resistance, sensitivity to endogenous hormones, abnormal hypothalamic-pituitary-adrenal axis function, nutritional deficiencies, alteration of glucose metabolism, and fluid and electrolyte imbalance [13,16-18].

Until recently, the focus on single, usually pharmacologic therapy has dominated the treatment of PMS. But now clinical research suggests that combination of treatments including pharmacotherapies (like selective serotonin reuptake inhibitors (SSRIs), anxiolytic agents, gonadotropin-releasing hormone (GnRH) agonists, the diuretic spironolactone, non-steroidal anti-inflammatory drugs and combination oral contraceptives (OCs)), cognitive and behavioral therapies, aerobic exercises, homeopathic remedies, reflexology, light therapy, massage therapy, dietary and nutritional modifications have been used over the years to treat premenstrual symptoms are more beneficial than are single treatments [19-25]. Hence, the primary aim of this study was to assess the prevalence, impacts and medical managements of PMS on female students of College of Health Sciences students, Mekelle University.

## Methods

The study was conducted in College of Health Sciences, Mekelle University, which is found in Mekelle, the capital city of Tigray region, located 783 kilometers north of Addis Ababa, the capital of Ethiopia [26]. A cross-sectional survey was conducted from March to April 2013 using a self-administered questionnaire among female students of College of Health Sciences, Mekelle University. The participants were given liberal verbal explanations plus description letters about the topic and the aim of the study. The sample size was determined by using single proportion for finite population with 95% confidence interval, marginal error (d) of 5% and the prevalence (P) of 50% and considering 10% non-response rate to be 258. The sampling technique used was systematic sampling for distributing the questionnaire to the individual participants and cluster sampling to group the participants according to their department and class year.

The study was conducted after getting approval from the Ethics Review Committee (ERC) of the College of Health Sciences, Mekelle University. Formal letter was written by the department of Pharmacy, Mekelle University to the student service center (Ref. No.: CHS/296/pharm05) and the letter was approved and permission was granted by the student service director. Participation was voluntary and no participant was enforced to answer the questionnaire. A written consent was taken from the participants after the aim of the study was explained for them. The participants were told that the information obtained from them will be kept with complete confidentiality and no attempt of abusing their information. For confidentiality purpose the names and addresses of the participants were not mentioned.

Inclusion criteria were:

1) Had a menstrual period at least in the last two consecutive months

2) Women within 15–49 years of age

3) Enrolled full-time in undergraduate studies

Exclusion criteria were:

1) Currently pregnant

2) History of chronic illness, diabetes, high blood pressure, heart disease, or current depression, anxiety, and any other psychiatric disorders

3) Currently using a hormonal method of contraception

4) Intern medical students, extension and in-service students

5) Has irregular menstrual cycle

A semi-structured and self-administered questionnaire was used for data collection. The questionnaire contained pertinent demographic characteristics of the study participants, their gynecologic and obstetrics profiles and possible symptoms of PMS assumed to be developed that were gathered from different literatures. Pretesting was done before starting of the study on 20 (7.75% of the calculated sample size) female students of Sheba University College in order to assess the validity and repeatability of the data collection instrument, highlight problems associated with the data collection tools, check the data collectors’ performance and ensure standardization of techniques; and the Cronbach alpha value was found to be 0.91. In this study, the criteria proposed by Diagnostic and Statistical Manual of Mental Disorders, fourth edition, text revision (DSM–IV TR) was used to assess the prevalence of PMS.

The collected data were checked for completeness and accuracy and corrected on daily basis before being filled. Data were coded and edited properly by the principal investigator prior to data entry. Before data analysis, data were cleaned and 5% of the data were re- entered to ensure data quality.

Data were analyzed using the Statistical Package For the Social Sciences, SPSS Inc., Chicago, IL (SPSS version 16). Descriptive analytical parameters were used to summarize the socio-demographic and clinical characteristics of the study participants. Summary tables, graphs and charts were used for descriptive purpose. The different socio-demographic, gynecologic and obstetric characteristics variables were presented, compared, analyzed and frequency distributions of the variables were interpreted. Chi-square test was used to assess the association of different factors with the PMS and the use of treatment protocols. Variables that were found to be associated with prevalence of PMS and the use of treatment protocols on this initial analysis by the Chi-square test were subject to multivariate logistic regression analysis model to assess the predictor variable(s). A p-value of < 0.05 was considered significant.

### Operational definitions

Mild PMS symptoms: Symptoms as minor as not interfering routine daily activities

Moderate PMS symptoms: Symptoms interfering routine daily activities

Severe PMS symptoms: Symptoms hindering participation in any activity

## Results

A total of 258 female students of college of health science, Mekelle University were enrolled into the study. The response rate was 86.43% which was 223 participants. But only 173 participants’ data were subjected for data analysis because 22 were incomplete, 14 were using contraceptives currently and 14 had irregular menstrual cycle. The mean age of the study participants was 20.86 ± 1.913 years. The mean height and weight of the study participants were 1.60 ± 0.076 meters and 51.77 ± 6.555 kilograms respectively. One hundred thirteen (65.3%) of the study participants were single and 52(30.1%) were in relationship (Table 1).

**Table 1:** **Socio**-**demographic**, **gynecologic and obstetric characteristics of study participants**, **Mekelle University College of Health Sciences**, **March**-**April 2013** (**n** = **173**)

**Characteristics**	**Frequency (N (%))**	**Mean (± standard error mean)**
Age		
<20	50(28.9)	20.86 ± 1.913
20-23	81(46.82)	
24-26	42(24.28)	
Height		
1.40-1.49	10(5.78)	1.60 ± 0.076
1.50-1.59	85(49.13)	
1.60-1.69	56(32.37)	
1.70-1.79	20(11.56)	
1.80-1.89	2(1.156)	
Weight		
≤45	30(17.34)	51.77 ± 6.555
45-55	96(55.49)	
56-65	40(23.12)	
≥66	7(4.046)	
Marital status		
Single	113(65.3)	
In relationship	52(30.1)	
Married	5(2.9)	
Divorced	1(0.6)	
Others	2(1.2)	
Class year of the students		
1st year	29(16.8)	
2nd year	53(30.6)	
3rd year	45(26)	
4th year	14(8.1)	
5th year and above	32(18.5)	
Department		
Generic nursing	21(12.1)	
Midwifery	12(6.9)	
Psychiatry nursing	8(4.6)	
Public health	24(13.9)	
Pharmacy	42(24.3)	
Medicine	62(35.8)	
Dental Medicine	4(2.3)	
Residence		
In the dorm	161(93.1)	
In private room	5(2.9)	
With family	7(4)	
Menarche		
<13	39(22.5)	
13-15	111(64.2)	
16-18	21(12.1)	
>18	2(1.2)	
Average length of one cycle of menstruation		
<28	19(11)	
28	100(57.8)	
>28	54(31.2)	
Number of days bleeding per one cycle		
1-3	47(27.2)	
4-5	99(57.2)	
6-8	25(14.5)	
>8	2(1.2)	
Menstrual flow type		
Mild	45(26)	
Moderate	109(63)	
Heavy	17(9.8)	
Extremely heavy	2(1.2)	

Among the study participants, 111(64.2%) started menstruation at the age of 13-15 years followed by the age of <13 years (39(22.5%)). The usual menstrual cycle of the participants was 28 days (100(57.8%)) and menstrual duration was 4–5 days (56.2%). The menstrual flow type of the majority of the participants were of moderate type which was 109(63.0%) followed by mild menstrual flow which was by 45(26.0%) (Table 1).

Among the participants, 144(83.2%) have PMS symptoms with their menstrual period.

The most commonly reported physical symptoms with PMS were abdominal bloating by 141(81.5%), abdominal cramps by 128(74.0%), breast tenderness by 118(68.2%), back pain by 115(66.5%), weakness by 107(61.9%), generalized body pain by 104(60.1%), and headache by 100(57.8%) of the participants, while the most commonly reported psycho-behavioral symptoms experienced by the participants were loss of interest in doing things which was by 134(77.5%), followed by depressed mood by 129(74.6%), anger feeling 99(57.2%), and difficulty concentrating 81(46.8%) (Table 2).

**Table 2:** **PMS symptoms among female students of Mekelle University College of Health Sciences**, **March**-**April 2013** (**n** = **173**)

**Types of symptoms**	**Behavior or characteristics**	**Degree of symptoms**			**Total**
		**Mild**	**Moderate**	**Severe**	
Physical or somatic symptoms	Abdominal bloating	87(50.3%)	41(23.7%)	13(7.5%)	141(81.5%)
	Breast tenderness	73(42.2%)	40(23.1%)	5(2.9%)	118(68.2%)
	Generalized body pain	50(28.9%)	38(22.0%)	16(9.2)	104(60.1%)
	Headache	51(29.5%)	37(21.4%)	12(6.9%)	100(57.8%)
	Back pain	33(19.1%)	60(34.7%)	22(12.7%)	115(66.5%)
	Weight gain	18(10.4%)	5(2.9%)	3(1.7%)	26(15.0%)
	Weight loss	19(11.0%)	8(4.6%)	1(0.6)	28(16.2%)
	Shortness of breath	26(15.0%)	14(8.1%)	2(1.2%)	42(24.3%)
	Abdominal cramps	52(30.1%)	51(29.5%)	25(14.4%)	128(74.0%)
	Weakness	38(22.0%)	50(28.9%)	19(11.0%)	107(61.9%)
	Vomiting	16(9.3%)	12(6.9%)	6(3.5%)	34(19.7%)
Emotional or psycho-behavioral symptoms	Eating more than usual	22(12.7%)	15(8.7%)	4(2.3%)	41(23.7%)
	Anger	45(26.0%)	28(16.2%)	26(15.0%)	99(57.2%)
	Loss of interest in doing things	57(33.0%)	45(26.0%)	32(18.5%)	134(77.5%)
	Depressed mood	53(30.6%)	43(24.9%)	18(19.1%)	129(74.6%)
	Craving for sweat foods and alcohol and appetite change	24(13.9%)	17(9.8%)	9(5.2%)	50(28.9%)
	Forgetfulness	20(11.6%)	13(7.5%)	3(1.7%)	36(20.8%)
	Sleep disturbances	29(16.8%)	21(12.1%)	6(3.5%)	56(32.4%)
	Difficulty concentrating	32(18.5%)	39(22.5%)	10(5.8%)	81(46.8%)

Eighty three (48.0%) of the participants reported academic performance impairment in the whole daily activities with their periods while the remaining reported that they did not have performance interference with the PMS. Among those reported performance impairment due to PMS (Table 3), 49(28.3%) reported frequent class missing, 17(9.8%) exam missing, 14(8.1%) low grade scoring associated with their PMS and 3(1.7%) of them reported withdrawal from their learning.

**Table 3:** **Impacts of PMS on female students of Mekelle University College of Health Sciences**, **March**-**April 2013** (**n** = **173**)

**Performance impairment**		**Frequency (N (%))**
Academic performance impairment	Yes	83(48.0)
	No	90(52.0)
Types of performance impairment	Frequent class missing	49(28.3)
	Exam missing	17(9.8)
	Low grade scoring	14(8.1)
	Academic withdrawal	3(1.7)
Scoring less than boys due to PMS	Yes	28(13.9)
	No	165(82.1)

**Figure 1:**
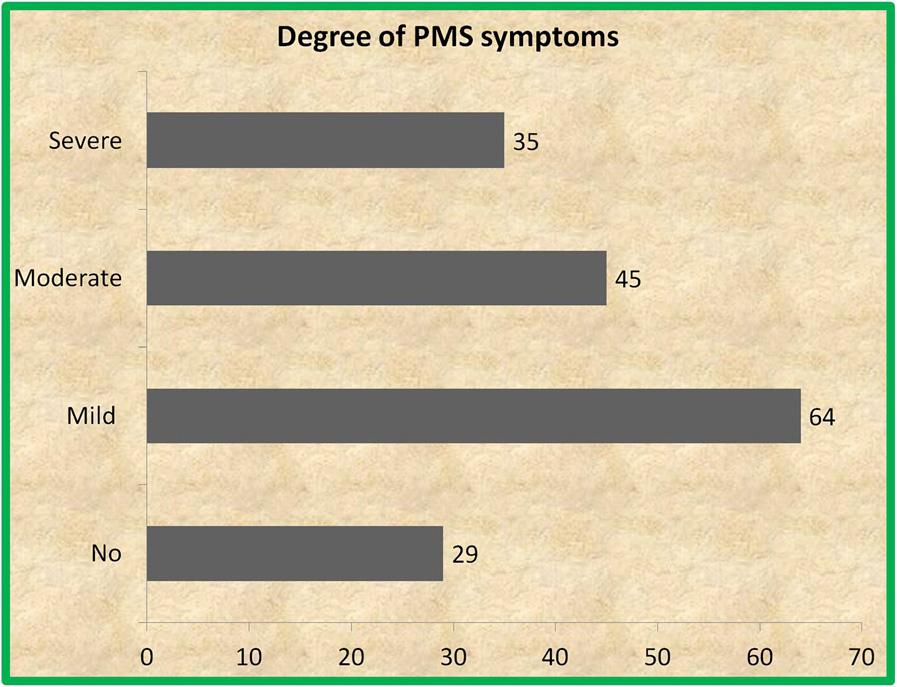
The degree of symptoms of PMS among female students of Mekelle University College of Health Sciences, March-April 2013 (n = 173).

**Figure 2:**
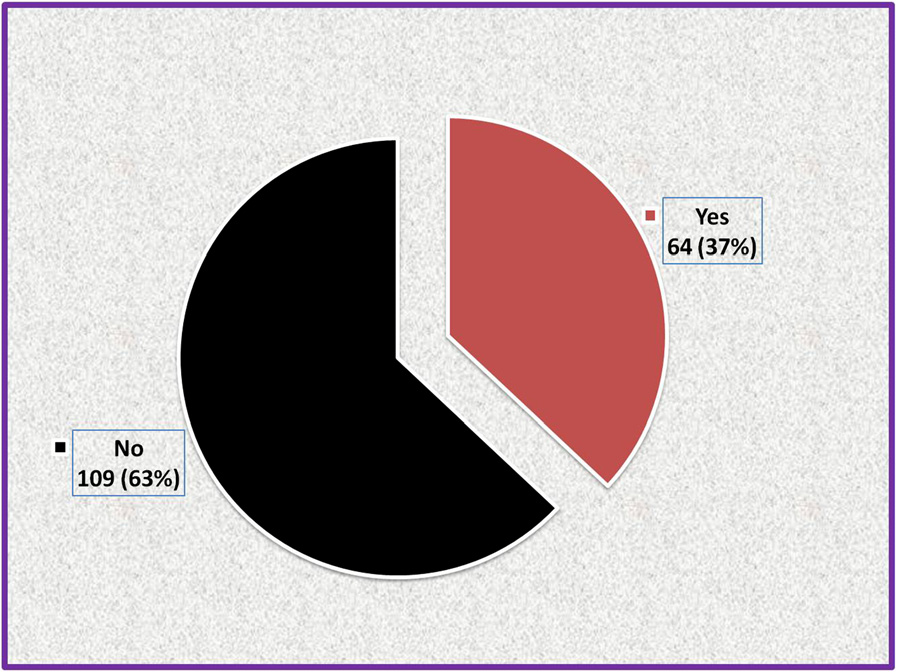
The prevalence of PMS in female students of Mekelle University College of Health Sciences, March-April 2013 (n = 173).

Sixty four (37%) of the participants had mild type of PMS symptoms; the second common type being of moderate type (45(26%)) (Figure 1). According the Diagnostic and Statistical Manual of Mental Disorders, fourth edition, text revision diagnostic criteria for PMS/premenstrual dysphoric disorder (DSM IV-TR), 64(37.0%) students fulfilled the diagnostic criteria for PMS (Figure 2).

Only 83(48.0%) participants sought medical treatment for their PMS symptoms. The common treatment modalities used were pain killers, 63(36.4%), hot drinks like coffee and tea, 13(7.5%), and massage therapy, 4(2.3%), (Table 4).

**Table 4:** **Types of PMS treatment and methods of coping with PMS among female students of Mekelle University College of Health Sciences**, **March**-**April 2013** (**n** = **173**)

**Treatment**	**Frequency (N (%))**	
Treated for PMS		
Yes	83(48.0)	
No	90(52.0)	
Types of PMS treatment		
Pain killers	63(36.4)	
Hot drinks like coffee and tea	13(7.5)	
Massage	4(2.3)	
Exercise and others	3(1.7)	

Under Pearson Chi-square, number of days of bleeding per one cycle of menstruation was significantly associated with both PMS prevalence and use of PMS treatment (Table 5, Table 6) and academic performance impairment was significantly associated with the use of PMS treatment (Table 6).

**Table 5:** **The association of demographic and gynecologic**/**obstetrics factors with PMS**, **March**-**April 2013** (**n** = **173**)

**Variables**	**Diagnosis of PMS**	
	***X***^***2***^	**P-value**
Marital status	6.365	0.173
Class year in university	7.370	0.118
Department	8.278	0.218
Residence	1.303	0.521
Chronic disease	.041	0.839
Past contraceptive use	.592	0.442
Age of first menstruation	1.501	0.682
Average length of one cycle of menstruation	3.824	.281
Number of days of bleeding per one cycle	13.102	.004*
Menstrual flow type	7.083	.069

Those denoted as ’*’ are said to have association with PMS for p < 0.05.

**Table 6:** **The association of demographic**, **gynecologic**/**obstetrics factors and PMS symptoms with the use of PMS treatments**, **March**-**April**, **2013** (**n** = **173**)

**Variables**	**Use of PMS treatments**	
	***X***^***2***^	**P-value**
Marital status	8.603	.072
Class year in university	4.735	.316
Department	7.818	.252
Residence	.365	.833
Chronic disease	.271	.603
Past contraceptive use	.937	.333
Number of year of contraceptive use	2.000	.157
Age of first menstruation	6.782	.079
Average length of one cycle of menstruation	2.104	.551
Number of days of bleeding per one cycle	9.438	.024*
Menstrual flow type	4.636	.200
Academic performance impairment	13.764	.000*
Type of performance impairment	1.157	.763
Abdominal bloating	5.190	.637
Breast tenderness	3.992	.551
Generalized body pain	2.412	.878
Headache	8.273	.309
Back pain	5.559	.592
Weight gain	7.803	.253
Weight loss	6.021	.304
Eating more than usual	5.546	.594
Shortness of breath	6.183	.519
Abdominal cramp	6.671	.464
Weakness	.712	.982
Vomiting	4.265	.512
Difficulty concentrating	5.738	.333
Sleep loss	5.438	.489
Forgetfulness	5.502	.358
Craving for sweet foods and alcohol	8.677	.277
Depressed mood	10.232	.176
Loss of interest in doing things	3.295	.856
Anger	11.306	.185

Those denoted as ’*’ are said to have association with the use of PMS treatment for p < 0.05.

Binary logistic regression analysis revealed that participants whose average length of one cycle of menstruation is 1–3 days were five times (COR = 0.20, 95% C.I = 0.070-0.569, P-value = 0.003) more likely to develop PMS as participants whose average length of one cycle of menstruation is 6–8 days. With similar analytical procedure, participants with academic performance impairment were three times (AOR = 0.345, 95% C.I = 0.183-0.653, P-value = 0.001) more likely to use PMS treatments as participants who do not have academic performance impairment.

## Discussions

We performed a survey in 18–25 years old health science students, whose characteristics were similar to previous study in 2002 from Ethiopia [6]. In our study, the prevalence of PMS was found to be 37.0%. This figure is almost the same with a research done in Tainan, Taiwan [26] with prevalence of PMS found to be 39.5%, but slightly lower than another study conducted at Isra University Hospital, Hyderabad, Sindh, Pakistan in 2008, on 172 participants [27] that the prevalence of the PMS was 51%. Another study done in Jimma University, in 2002 [6] on 242 female students showed the prevalence of PMS to be 27% which is slightly lower than that of this study. This slight difference may be due to the fact that the previous study done in Jimma University included both the social and health sciences students. But in our study only health sciences students were included which are all the time in academic stress leading to the high prevalence of PMS. Secondly we have used smaller sample size than the study done in Jimma University. This study is in agreement with the study done among female students of Assumption University in Bangkok, Thailand on 266 female students which showed that the prevalence of PMS to be 28% [2] and another study performed at the College of Medicine, King Faisal University, Saudi Arabia on 250 students which showed that PMS was diagnosed in 89 (35.6%) of participants using the ACOG criteria [28].

As it is illustrated in Table 2, the most commonly reported physical symptoms with PMS were abdominal bloating, 141(81.5%), and the most commonly reported psycho- behavioral symptoms experienced by the participants was loss of interest in doing things 134(77.5%,) while in other study done in Jimma University, the commonest psycho-behavioral PMS symptom was decreased interest in the usual activities affecting 177(73.1%) and the commonest in the physical symptom group was easy fatigability affecting 170(70.2%) [6]. According to research done in Jinnah Medical & Dental College Karachi from July 2009 to September 2009 [9], the most prevalent symptoms among the medical students all above 50%, were increased appetite (67.5%), worry and anxiety (60%), tired or lethargic (54%) felt suddenly sad/tearful (56.5%), interpersonal conflict (54%) and depressed mood (52.5%). The research done in College of Medicine, King Faisal University, Saudi Arabia, from June through December 2009 [28], also showed that the most frequently reported symptom was abdominal bloating (75.3%).

In our study, the most commonly prevalent performance impairment interfering with the daily activities of the participants was frequent class missing, 49(28.3%) and exam missing 17(9.8%), (Table 4). This is in agreement with the study done in Iran in which 80 (25%) of participants missed the classes and examinations leading to decline in education [29]. Another study done in Saudi Arabia [28] reported that performance impairment like poor concentration in class (48.3%), low college attendance (46%), going out of the home (43.8%), daily home chores (41.6%) and homework tasks (36%). It is predictable that these students suffering from PMS after graduating and getting a job, would be periodically absent at work and have reduced productivity. Several researches have shown that PMS can have both direct and indirect economic consequences [1,10,12]. Another study done in SRM University, Kattankulathur, Tamilnadu, India showed that up to 40% of female students in the study reported that their ability to perform work was affected [30].

Concerning remedy usage and methods of PMS treatment, 83(48.0%) of the participants reported that they have sought medical treatment for their PMS. The remaining 90(52.0%) did not treated for their symptoms. But 83.20% of the participants reported PMS symptoms with their menses. This implies that around half of the participants remain untreated. This may be not because they do not have the problem or the problem is not interfering with their daily activities, but either because they might be fearful to seek treatment for menstrual and related problems due to cultural or other reasons [6], or treatment facility was not readily available. Pain is often disregarded by many women who consider pain to be a normal part of the menstrual cycle [30]. Thus, many women fail to report their pain to physicians. The problem of absenteeism from school or work was also under- appreciated. The common type of treatment used by participants with PMS symptoms were pain killers like aspirin and ibuprofen 63(36.4%) and hot drinks like coffee and tea 13(7.5%); the others being massage therapy, yoga, exercise and applying heat around pelvic area. Research done in US also shows that the commonly used remedy was ‘antipain’ and these medicines were the most commonly prescribed in the survey done in US [31]. Sodium restriction has been proposed to minimize bloating, fluid retention, and breast swelling and tenderness. On the contrary, caffeine restriction was recommended because of the association between caffeine and premenstrual irritability and insomnia [5].

Participants whose average length of one cycle of menstruation is 1–3 days were five times more likely to develop PMS as participants whose average length of one cycle of menstruation is 6–8 days as binary logistic regression analysis revealed. This could be explained by the light and shorter duration of menses associated with rapid fluctuations of estrogen and progesterone and hence, development of PMS symptoms during the late luteal phase which are responsible for endometrial growth as its growth status determines the volume and duration of menses. With the same approach, participants with academic performance impairment were three times more likely to use PMS treatments as participants who do not have academic performance impairment which could be due to the fact that participants with academic performance impairments might be more concerned with the PMS symptoms with the perception that the impairment would let them to drop out or fail down from their academia unless they are treated well. On the contrary, the only socio-demographic factor associated with severity and treatment of PMS in Thailand Assumption University is age [2]. The possible reason why this could not be associated in our study could be the narrow age gap among our study participants. But, the American Academy of Family Physicians and the American College of Obstetrics and Gynecology recommend adequate sleep as one of the lifestyle alterations used to treat PMS [9]. The variations of results and estimates of PMS from various studies could be due to limitations and differences in the definition of PMS, standards and methods of data collection, sampling technique, type of patient population studied and differences in instruments, symptom’s patterns, the number of symptoms reported and the use of prospective or retrospective protocols. In addition, the research literature does not agree as to the number of symptoms that must be present to warrant a diagnosis of PMS. However, in spite of these inconsistencies, it is apparent from the sample population of this study that the reproductive age of women in health sciences students of Mekelle University suffer from PMS and it interferes with daily functioning among 37.0% of the respondents.

### Limitations of the study

This study was limited to college of health sciences students of Mekelle University in Ethiopia and does not represent the whole female university student population in Ethiopia. Since the topic is sensitive for the Ethiopian culture, some respondents might not want to reveal their real personal problems. The use of retrospective questionnaires is not the best method for data collection of PMS symptoms as the ideal way is by the prospective logging of symptoms by the respondents over at least two cycles. But the investigators couldn’t conduct it prospectively as a matter of budget constraint as the study was undertaken without financial support from any sources.

## Conclusions

The prevalence of PMS in general is high among health sciences students of Mekelle University with prevalence of 37.0%. The most common physical PM symptom is abdominal bloating and the commonest psycho-behavioral symptom is loss of interest in doing things. Severe symptoms have negative impact on academic and social performances of the students by causing frequent class missing, exam missing, low grade scoring and academic withdrawal, which affect the life of the subjects and as the whole the country. Average number of days per each menses is found to affect the development of PMS and academic performance impairment is found to affect the use of treatment protocols for PMS.

## Abbreviations

ACOG: American College of Obstetrics and Gynecology; AOR: Adjusted odds ratio; COR: Crude odds ratio; DSM-IV TR: Diagnostic and Statistical Manual of Mental Disorders, fourth edition, text revision; GABA: Gamma amino butyric acid; GnRH: Gonadotropin releasing hormone; ICD: International Classification of Diseases; OCs: Oral contraceptives; PMDD: Premenstrual dysphoric disorder; PMS: Premenstrual syndrome; SPSS: Statistical Package for Social Sciences; SSRIs: Selective serotonin receptor inhibitors; US: United State.

## Competing interests

The authors declare that they have no competing interests.

## Authors’ contribution

FWT: Have made substantial contributions to conception and design, or acquisition of data, or analysis and interpretation of data. MLB: Have made substantial contributions to conception and design, or acquisition of data, or analysis and interpretation of data. Have been involved in drafting the manuscript or revising it critically for important intellectual content; and have given final approval of the version to be published. Both authors read and approved the final manuscript.

## Pre-publication history

The pre-publication history for this paper can be accessed here:

http://www.biomedcentral.com/1472-6874/14/52/prepub
